# Comparison of CTX-M encoding plasmids present during the early phase of the ESBL pandemic in western Sweden

**DOI:** 10.1038/s41598-024-62663-2

**Published:** 2024-05-24

**Authors:** Moa S. Wranne, Nahid Karami, K. K. Sriram, Daniel Jaén-Luchoro, Shora Yazdanshenas, Yii-Lih Lin, Arpitha Kabbinale, Carl-Fredrik Flach, Fredrik Westerlund, Christina Åhrén

**Affiliations:** 1 Department of Life Sciences, Chalmers University of Technology, Gothenburg, Sweden; 2 Department of Infectious Diseases, Institute of Biomedicine, University of Gothenburg, Guldhedsgatan 10A, 413 46 Gothenburg, Sweden; 3 Department of Clinical Microbiology, Sahlgrenska University Hospital, Region Västra Götaland, Gothenburg, Sweden; 4 Centre for Antibiotic Resistance Research in Gothenburg (CARe), University of Gothenburg, Gothenburg, Sweden; 5 Swedish Strategic Program Against Antimicrobial Resistance (Strama), Region Västra Götaland, Gothenburg, Sweden

**Keywords:** Microbiology, Antimicrobials, Antimicrobial resistance

## Abstract

Plasmids encoding *bla*_CTX-M_ genes have greatly shaped the evolution of *E. coli* producing extended-spectrum beta-lactamases (ESBL-*E. coli*) and adds to the global threat of multiresistant bacteria by promoting horizontal gene transfer (HGT). Here we screened the similarity of 47 *bla*_CTX-M_ -encoding plasmids, from 45 epidemiologically unrelated and disperse ESBL-*E. coli* strains, isolated during the early phase (2009–2014) of the ESBL pandemic in western Sweden. Using optical DNA mapping (ODM), both similar and rare plasmids were identified. As many as 57% of the plasmids formed five ODM-plasmid groups of at least three similar plasmids per group. The most prevalent type (28%, IncIl, pMLST37) encoded *bla*_CTX-M-15_ (n = 10), *bla*_CTX-M-3_ (n = 2) or *bla*_CTX-M-55_ (n = 1). It was found in isolates of various sequence types (STs), including ST131. This could indicate ongoing local HGT as whole-genome sequencing only revealed similarities with a rarely reported, IncIl plasmid. The second most prevalent type (IncFII/FIA/FIB, F1:A2:B20) harboring *bla*_CTX-M-27_, was detected in ST131-C1-M27 isolates, and was similar to plasmids previously reported for this subclade. The results also highlight the need for local surveillance of plasmids and the importance of temporospatial epidemiological links so that detection of a prevalent plasmid is not overestimated as a potential plasmid transmission event in outbreak investigations.

## Introduction

Bacteria producing extended-spectrum beta-lactamases (ESBLs) are generally multidrug resistant and pose a serious challenge to our healthcare systems^1^. The most prevalent ESBL-producing bacteria are ESBL-producing *Escherichia coli* (ESBL-*E. coli*). They mostly cause urinary tract infections (UTIs), but also severe infections, such as septicaemia^2^. *E. coli* has an extensive phylogenetic substructure and is divided into seven phylogroups (A, B1, B2, C, D, E and F) that correspond with sequence types (STs)^3,4^. The number of STs associated with ESBL- *E. coli* is steadily increasing, but certain STs are more prevalent than others and are often referred to as high-risk clones^5^. Among these, ST131 accounts for the greatest share of multidrug-resistant extraintestinal *E. coli* infections in human worldwide^6–8^. Three major clades, A, B and C, have been described within ST131^8–10^. Clade C includes three sublineages C0, C1 and C2 where C0 may be an evolutionary precursor to the two dominating linages C1 and C2. The C1-M27 sublineage has emerged from C1 in more recent years and is becoming increasingly prevalent^11^.

The most prevalent ESBLs in *E. coli* are those encoded by *bla*_CTX-M_ genes, primarily those within CTX-M-group 1 (especially *bla*_CTX-M-3_, *bla*_CTX-M-15_ and *bla*_CTX-M-55_) and CTX-M-group 9 (especially *bla*_CTX-M-14_ and *bla*_CTX-M-27_), but with geographical and temporal differences^12^. The *bla*_CTX-M_ genes are often found on conjugative plasmids, which facilitates their spread by horizontal gene transfer (HGT)^8,13,14^. Plasmids part of incompatibility (Inc) group F are the most common plasmids in ESBL-*E. coli* causing human infections, followed by IncIl plasmids. Numerous types of plasmids have been reported, with geographical differences and local predominance of certain types^13,15,16^. Some plasmids with *bla*_CTX-M_ genes are shared by multiple, unrelated clones, whereas some clones appear to be very closely connected to certain plasmids, suggesting a stable and fixed coevolution of plasmids, clones and subclones worldwide. This phenomenon has primarily been explored for IncF-plasmids and ST131 and rarely for other plasmids and clones^13,14,17,18^. Additionally, a strong association between IncF plasmids in general and ST131 has been described, especially for IncFIA-FII, FIA-FIB-FII and FIB-FII plasmids^14–16^.

In epidemiological studies of plasmids, replicon typing into various Inc groups has traditionally been used. With the introduction of sequencing methods, plasmids are being increasingly organized into plasmid multi-locus sequence types (pMLSTs)^19^. For IncF plasmids, a replicon sequence typing (RST) scheme is generally used to identify FII, FIA, and FIB (FAB-formula) types^20^. For even more detailed information and comparisons of plasmids, whole-genome sequencing (WGS) methods are increasingly used. Another useful method for plasmid characterization is optical DNA mapping (ODM), which we have used in several studies to characterize and compare plasmids encoding antibiotic resistance genes^21–24^. In the ODM protocol, we use competitive binding between YOYO-1 and netropsin to create a sequence dependent intensity variation (a barcode) along the DNA. The DNA is then stretched using nanochannels and imaged with a fluorescence microscope on the single DNA molecule level^25^. CRISPR/Cas9 is used to identify and locate the resistance gene of interest^26^. With this method, full-length plasmids can be characterized at the single plasmid level. This technology provides long-range sequence information that correlates well with long-read WGS results^27^. By constructing theoretical barcodes from closed long-read WGS sequence data, it is possible to compare an experimental barcode to the theoretical barcode of a plasmid already characterized by WGS^28^.

The local establishment and spread of multidrug resistant bacteria vary greatly and depend on the features of a given setting^29^. In western Sweden, ESBL-*E. coli* was first detected in 2003 and then established slowly reaching approximately 2% of clinically detected *E. coli* in 2008^30^ and 3% by 2014. During this time-period CTX-M group 1 genes, especially *bla*_CTX-M-15_, became dominant and isolates expressing CTX-M enzymes constituted a very polyclonal set of strains. Additionally, a recent study of ours^22^, somewhat surprisingly, revealed similarities between *bla*_CTX-M_ encoding plasmids isolated from a limited number of epidemiologically unrelated UTI isolates in western Sweden. Taken together, these findings could indicate ongoing HGT of plasmids encoding *bla*_CTX-M_ genes already at the early stage when ESBL-*E. coli* were established in our region.

To further elaborate on these findings, we here used ODM to investigate the plasmid similarity of CTX-M-encoding plasmids from a larger set of highly disperse ESBL-*E. coli* strains with no existing epidemiological relationships. They were isolated both from the hospital and the community setting during 2009–2014 in western Sweden. The aim was to understand whether there were few successful plasmids in circulation at the time, or if many different plasmids encoding *bla*_CTX-M_ genes were present. In the case of outbreak investigations, this may be important knowledge to not overestimate a prevalent plasmid as a potential transmission event.

## Results

In this study, we included ESBL-*E. coli* isolates from 45 patients of different sexes, ages and levels of care (Tables 1 and 2). The bacteria were isolated from clinical urinary samples (n = 33) and fecal screen samples (n = 12). The isolates were arbitrarily selected from a larger collection of previously typed strains isolated in our region during the study-period (as detailed in the methods) and we aimed for including isolates of diverse strain types harbouring *bla*_CTX-M_ genes of the most dominant CTX-M groups, i.e., group 1 and 9.

**Table 1:** Characteristics of *E. coli* isolates and plasmids with CTX-M group 1 genes.

Plasmid ID	Time (year)	Patient sex/age (year)	Type	HC/PC	ST/ST131-subclade	CC	Phylo-group	Replicon type (Inc)	Size (kb)	ODM-plasmid group	Ref
15-1	2010	F/59	U	PC	131-C0	131	B2	FII, FIA, FIB, I1	93	A	^22^
15-2	2009	F/20	U	PC	2141	None	F	FII, FIA, FIB, I1	89	A	^22^
15-3	2009	F/82	U	PC	131-C0	131	B2	FII, FIA, FIB, I1	87	A	^23^
15-4	2010	F/50	U	HC	167	10	A	FII, I1	87	A	t.s
15-5	2010	F/71	U	HC	12	12	B2	FII, I1	94	A	^22^
15-6	2010	F/35	S	–	1193	14	B2	FII, FIA, FIB, I1	93	A	t.s
15-7	2011	F/41	S	–	517	469	B1	FIB, I1	93	A	t.s
15-8	2012	M/1	U	PC	131-C1	131	B2	FIA, FIB, FII	98	A	^22^
15-9	2012	M/9	S	–	2076	394	D	FII, FIB, I1	89	A	t.s
15-10	2014	F/32	S	–	131-A	131	B2	FII, FIB, I1	93	A	t.s
15-11	2009	F/20	U	PC	131-A	131	B2	FII, FIA, FIB	73	C	^22^
15-12	2011	F/2	U	HC	394	394	D	FII, K/B	95	–	t.s
15-13	2012	M/1	U	HC	131-C1	131	B2	FII, FIA, FIB	96	–	t.s
15-14	2012	M/57	U	HC	88	23	B1	N	93	–	^23^
15-15	2012	F/72	U	HC	315	38	D	FII, Y	73	–	t.s
15-16	2012	F/34	S	–	62	None	F	FII, FIB, P, B/O	89	–	t.s
15-17a	2014	F753	S	–	69	69	D	FII, I1	98	–	t.s
15-17-b	2014	F/53	S	–	69	69	D	FII, I1	99	–	t.s
15-18	2014	F/83	U	HC	131-C2	131	B2	FII, FIA, FIB, N, I1	93	–	t.s
15-19	2009	F/82	S	–	141	None	B2	–	134	B	t.s
15-20	2010	F/92	U	HC	167	10	A	FII, FIA, FIB	151	B	t.s
15-21	2013	F/85	U	HC	617	10	A	FII, FIA, FIB	159	B	^22^
15-22	2009	F/59	U	PC	131-B1	131	B2	FII, FIA, FIB, I1, B/O	189		^22^
15-23	2010	F/61	S	HC	1598	None	A	FIB	111	–	t.s
15-24	2011	M/73	U	HC	131-A	131	B2	FII, FIB	156	–	t.s
3-1	2010	M/33	U	PC	354	354	F	FII, FIA, FIB	71	C	^22^
3-2-a	2013	F/25	S	–	10	10	A	FII, I1	70	C	t.s
3-2-b	2013	F/25	S	–	10	10	A	FII, I1	88	A	t.s
3-3	2014	M/59	U	PC	4038	None	B1	I1	99	A	^23^
55-1	2010	M/33	U	HC	10	10	A	FII, FIA, FIB, N, I1	86	–	t.s
55-2	2014	M/70	U	PC	1193	14	B2	FII, FIA, FIB, Y, I1	90	A	t.s

The first number in the plasmid ID refers to the specific *bla*_CTX-M_-gene.

The letters -a and -b refers to two different plasmids detected in the same isolate. Plasmid 15-1 (pMLST 37) was chosen as reference for ODM-plasmid group A.

*HC/PC* clinical samples from patients in hospital care/primary care, *U* clinical urine samples, *S* fecal screen samples, *F* females, *M* male patients, *ST* sequence type, *CC to ST* clonal complex, *Ref* reference number in the reference list for previously reported plasmids, *t.s.* this study.

**Table 2:** Characteristics of *E. coli* isolates and plasmids with CTX-M group 9 genes.

Plasmid ID	Time (year)	Patient sex/age (year)	Type	HC/PC	ST/ST131-subclade	CC	Phylo-group	Replicon type (Inc)	Size (kb)	ODM group	Ref
27-1	2011	M/15	S	–	131-C1-M27	131	B2	FII, FIA, FIB	111	D	t.s
27-2	2011	F/46	U	HC	131-C1-M27	131	B2	FII, FIA, FIB, I1	82	D	t.s
27-3	2013	F/70	U	HC	131-C1-M27	131	B2	FII, FIA, FIB	135	D	t.s
27-4	2014	M/72	S	–	131-C1-M27	131	B2	FII, FIA, FIB, I1	150	D	t.s
27-5	2014	M/70	U	HC	131-C1-M27	131	B2	FII, FIA, FIB, I1	168	D	t.s
27-6	2008	M/65	U	PC	131-A	131	B2	FII, FIA, FIB, N	70	–	^22^
27-7	2008	M/65	U	PC	141	None	B2	FII, N	86	–	^22^
27-8	2009	M/14	U	PC	10	10	A	FII	92	–	^23^
27-9	2011	F/34	U	PC	58	155	B2	FII, FIA, FIB, I1, Y, B/O	102	–	^22^
27-10	2012	F/34	U	PC	421	95	B1	FIB, FII, B/O	114	–	^22^
27-11	2014	M/55	U	HC	131-C1-M27	131	B2	FII, FIA, FIB, I1	94	E	t.s
14-1	2009	M/2	U	PC	131-C1	131	B2	FII, FIA, FIB, I1	104	–	^22^
14-2	2010	F/71	U	PC	69	69	D	FII, FIB, B/O	93	–	^22^
14-3	2014	F/69	U	HC	131-A	131	B2	FII, FIA, FIB	94	E	t.s
14-4	2014	F/44	S	–	131-A	131	B2	FII, FIA, FIB	129	E	t.s
14-5	2012	F/80	U	HC	38	38	D	FII	47	–	t.s

The first number in the plasmid ID refers to the specific *bla*_CTX-M_-gene.

Plasmid 27-1 (FAB-formula F1:A2:B20) was chosen as reference for ODM-plasmid group D.

*HC/PC* clinical samples from patients in hospital care/primary care, *U* clinical urine samples, *S* faecal screen samples, *F* female, *M* male patients, *ST* sequence type, *CC to ST* clonal complex, *Ref* reference number in the reference list for previously reported plasmids, *t.s.* this study.

For most of the isolates, multiple plasmid replicon types were identified. Among all but two isolates, plasmids with replicon types of the IncF-family were identified, and 22 isolates carried plasmids with IncI1 replicons. In the ODM-analysis, only plasmids cut by CRISPR/Cas9 were included. i.e. plasmids encoding a *bla*_CTX-M_ gene belonging to group 1 or 9. To evaluate the similarity between two plasmids in the ODM analysis, their barcodes were compared using a p-value principle as described previously^24^. If the p-value was 0.01 or lower, the compared barcodes, and hence plasmids, were considered similar. If three or more barcodes were similar to each other, the plasmids were grouped together in an ODM-plasmid group. Five such groups (denoted A-E) were identified, as outlined below.

### ODM-groups for plasmids encoding CTX-M group 1 genes

**Figure 1:**
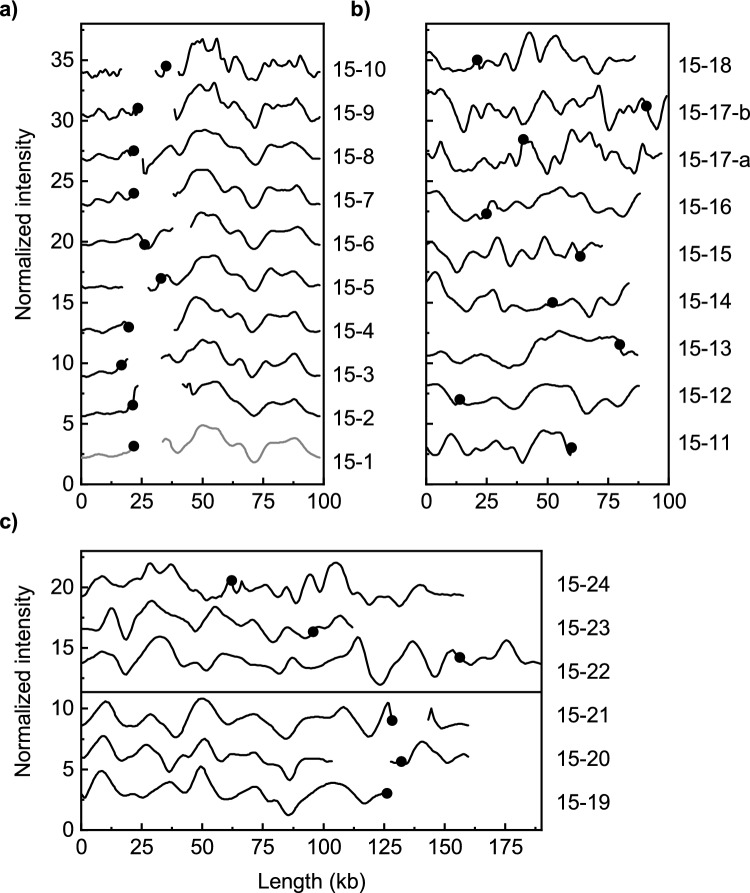
Comparison of ODM-barcodes for plasmids harboring the *bla*_CTX-M-15_ gene. The plasmids are grouped based on similarity and length and the plasmids from the most recent isolates are at the top of each subfigure. The black dots indicate the location of the *bla*_CTX-M-15_ gene. (**a**) Plasmids shorter than 100 kb with similar barcodes (ODM-plasmid group A). (**b**) Plasmids shorter than 100 kb with barcodes not fulfilling the criterion to be included in ODM-plasmid group A. (**c**) Plasmids longer than 100 kb. Barcodes with high similarity at the bottom (ODM-plasmid group B) and barcodes with low similarity at the top of the subfigure.

In most isolates only one plasmid encoding a *bla*_CTX-M_ gene was identified, but in two isolates two different plasmids encoding the same *bla*_CTX-M_ gene (*bla*_CTX-M-15_ and *bla*_CTX-M-3_, respectively) were found (Table 1). Nineteen of 25 plasmids encoding *bla*_CTX-M-15_ had a length of 73–99 kb (average length 92 kb) (Table 1). Ten of these were grouped together based on the similarity criterion (ODM-plasmid group A, Fig. 1a). Plasmid 15-1 was chosen as the ODM-reference plasmid for this group. While the plasmid barcodes were similar for all the plasmids, there were some smaller deletions/insertions around the location of the *bla*_CTX-M-15_ gene in some of the plasmids and the location of the *bla*_CTX-M-15_ gene varied slightly (Fig. 1a). The remaining nine shorter *bla*_CTX-M-15_ plasmids had barcodes that did not fulfill the criteria for inclusion in ODM-plasmid group A (Fig. 1b). Among the longer *bla*_CTX-M-15_-encoding plasmids, 111–189 kb (average length 150 kb) three similar plasmids were found (ODM-plasmid group B, Fig. 1c). The variation in length (134–159 kb) within this group indicated that some insertion/deletion events may have occurred also in these plasmids.

**Figure 2:**
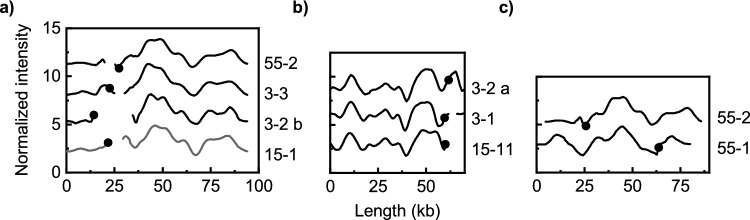
Comparison of ODM-barcodes for plasmids harboring the *bla*_CTX-M-3_ or *bla*_CTX-M-55_ gene. The plasmids are grouped based on similarity and the plasmids from the most recent isolates are at the top of each subfigure. The black dots indicate the location of the CTX-M gene. (**a**) One *bla*_CTX-M-55_ (55-2) and two *bla*_CTX-M-3_ (3-2-b and 3-3) encoding plasmids with barcodes similar to those of ODM-plasmid group A, here represented by plasmid 15-1. (**b**) Two *bla*_CTX-M-3_-encoding plasmids (3-1 and 3-2-a) have barcodes similar to the *bla*_CTX-M-15_ plasmid 15-11 (ODM-plasmid group C). (**c**) The two *bla*_CTX-M-55_-encoding plasmids with nonsimilar barcodes.

We next analyzed the plasmids encoding *bla*_CTX-M-3_. Despite the low number of such plasmids (n = 4), two pairs of plasmids with similar barcodes and very similar *bla*_CTX-M_ gene location were found (Fig. 2a, b). The plasmids in one of these pairs (3-2-b and 3-3) were also similar to 55–2 (encoding *bla*_CTX-M-55_) and to all the plasmids in ODM-plasmid group A and thus included in this group. The other pair of plasmids with *bla*_CTX-M-3_ (3-1 and 3-2-a) were similar to one *bla*_CTX-M-15_-encoding plasmid (15-11), forming ODM-plasmid group C (Fig. 2b). The two plasmids encoding *bla*_CTX-M-55_, i.e. plasmids 55-1 and 55-2 had non similar barcodes (p-value = 0.020, Fig. 2c).

### ODM-groups for plasmids encoding CTX-M group 9 genes

**Figure 3:**
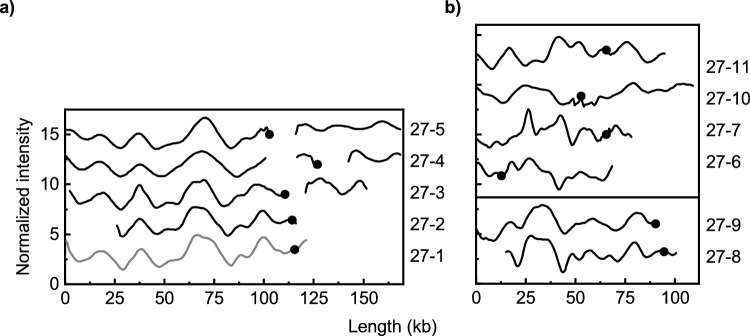
Comparison of ODM-barcodes for plasmids harboring the *bla*_CTX-M-27_ gene. The plasmids are grouped based on similarity and the plasmids from the most recent isolates are at the top of each subfigure. The black dots indicate the location of the CTX-M gene (**a**) *bla*_CTX-M-27_-encoding plasmids with similar barcodes (ODM-plasmid group D). (**b**) *bla*_CTX-M-27_-encoding plasmids not included in the ODM-plasmid group D. Two with similar barcodes (27-8 and 27-9) are shown at the bottom and the rest had nonsimilar barcodes.

We next investigated plasmids encoding genes belonging to CTX-M group 9 (Table 2). Five of the eleven *bla*_CTX-M-27_-encoding plasmids showed high similarity and were grouped to ODM-plasmid group D with 27-1 serving as the ODM-reference plasmid for this group (Fig. 3a). Interestingly, the plasmids were very different in length (82–168 kb) with an average length of 129 kb. Longer plasmids were found in more recent isolates, suggesting that new elements have been inserted into the plasmids over time. Among the remaining six plasmids encoding *bla*_CTX-M-27_, two had similar barcodes (27-8 and 27-9) while the others had low similarity (Fig. 3b).

**Figure 4:**
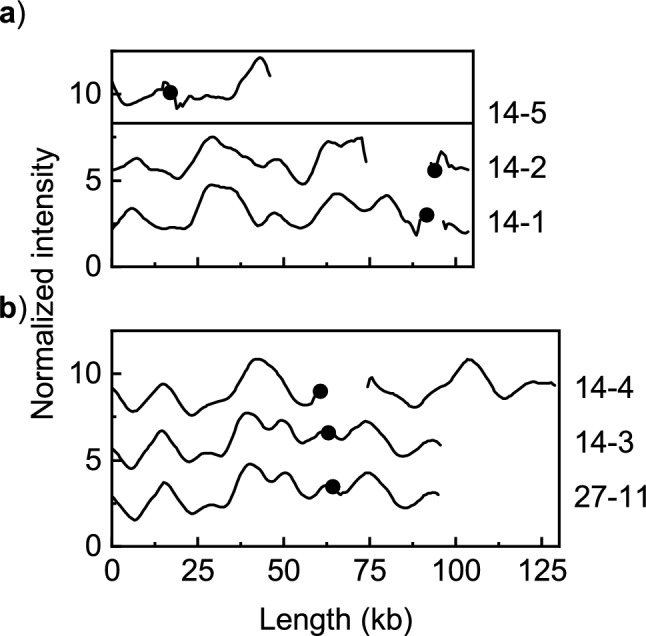
Comparison of ODM-barcodes for plasmids harboring the *bla*_CTX-M-14_ gene. The plasmids are grouped based on similarity and the plasmids from the most recent isolates are at the top of each subfigure. The black dots indicate the location of the CTX-M gene. (**a**) Plasmid 14-1 is similar to 14-2. Plasmid 14-5 is not similar to any of the other plasmids encoding *bla*_CTX-M-14_. (**b**) Plasmids 14-3 and 14-4 are similar to each other and to plasmid 27-11, which encodes *bla*_CTX-M-27_ (ODM-plasmid group E).

A comparison of the five isolates encoding *bla*_CTX-M-14_ revealed that 14-1 was similar to 14-2 (Fig. 4a) and 14-3 to 14-4 (Fig. 4b). Plasmid 14-5 showed no similarities with the other plasmids (Fig. 4a). Furthermore, plasmids 14-3 and 14-4 were found to be similar to plasmid 27-11 with *bla*_CTX-M-27_ forming ODM-plasmid group E. The plasmids in groups D and E did not fulfill the criteria for forming one large group but had similar features that suggest they might be related.

### Plasmid similarities in relation to sequence type

Plasmids from isolates that were part of the same ST were compared. The four ST10 isolates and the three ST69 isolates did not harbor similar plasmids. For the two ST1193 isolates, the plasmids (15-6, 55-2) were similar to each other, both being part of ODM-plasmid group A (Table 1, Figs. 1a and 2a), even though they encode different *bla*_CTX-M_ genes (*bla*_CTX-M-15_ and *bla*_CTX-M-55_). In addition, two of the plasmids (15-20 and 15-21) in ODM-plasmid group B (Table 1, Fig. 1c) were found in isolates of the same ST clonal complex (CC10).

The 19 ST131 isolates were further split into smaller groups based on their ST131 sublinages and some similarities were found. Two ST131-C0 isolates, one ST131-C1 isolate, and one ST131-A isolate all harbored plasmids belonging to ODM-plasmid group A (Table 1, Fig. 1a). Five of the six plasmids from the ST131-C1-M27 isolates were also similar (ODM-plasmid group D, Table 2, Fig. 3a) and there was only one ST131-C1-M27 isolate (27-11) with a *bla*_CTX-M-27_-encoding plasmid that did not belong to ODM plasmid group D. Finally, the *bla*_CTX-M-14_ encoding plasmids in the two ST131-A isolates were similar (Table 2, Fig. 4b).

### Comparison with theoretical ODM-barcodes constructed from reported WGS data

**Figure 5:**
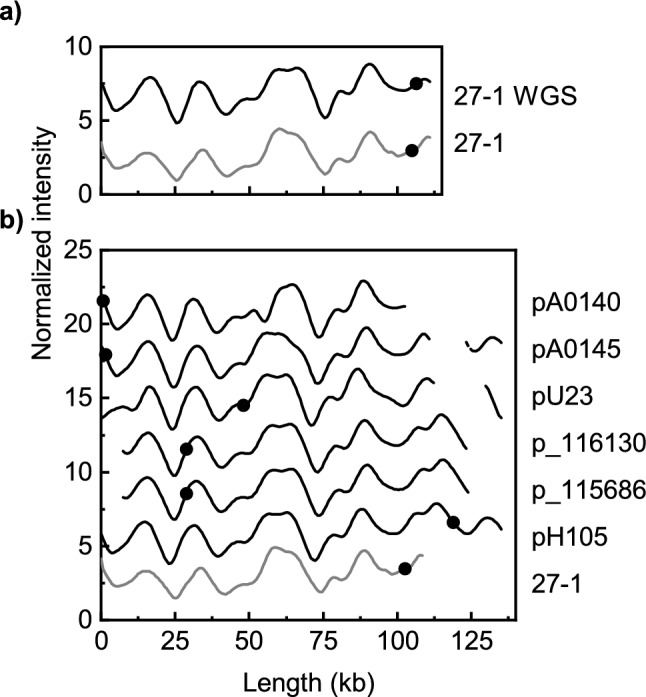
Comparison of the ODM-barcode for 27-1 with theoretical barcodes constructed from WGS data. (**a**) Comparison of the experimental ODM barcode from 27-1 with the theoretical barcode constructed from WGS of the 27-1 plasmid. (**b**) Comparison with reported F1:A2:B20-plasmids in ST131-C1-M27 isolates as detailed in Supplementary Table 1.

To further evaluate the epidemiology of the studied plasmids, we constructed theoretical plasmid ODM-barcodes from reported whole-genome sequence data for comparison with the experimental ODM-barcodes^28,31^. The primary interest was the two most prevalent plasmid types in the study, represented by the 15-1 and 27-1 plasmids. Plasmid 27-1 (representing ODM-group D) showed high similarity with theoretical barcodes constructed from WGS data of reported plasmids of similar length in ST131-C1-M27 isolates, including the pH105 plasmid from 2010 which is an often used reference for this type of plasmids (Fig. 5a, b)^17,32–34^. However, as shown in Fig. 5b, the gene locations were not identical for all the plasmids.

Subsequently, we searched the literature for completely sequenced plasmids encoding *bla*_CTX-M-15,_ both among commonly used reference plasmids^14,35–37^ and studies reporting local epidemiological data, including another Swedish study^13,17,18,38^. Unfortunately, studies from the same time-period mostly reported short read sequence data for IncF plasmids in ST131 isolates and often only on limited parts of the plasmids resulting in nonconclusive results. We extended the comparison to include all our plasmids encoding *bla*_CTX-M-15_ found in ST131 isolates. Plasmid 15-11 and the other two plasmids in ODM-plasmid group C, were found to be similar to the often-used reference plasmid c15-1a^35^. No other similarities were found for the plasmids encoding *bla*_CTX-M-15_ in the ST131 isolates in this study when compared with plasmids reported in the above studies (Supplementary Fig. S1). Finally, some similarities were found between plasmid 15-1 and the IncIl-plasmid pEK204 previously described by Woodford et al.^37^ (Supplementary Fig. S2). This could suggest that plasmid 15-1 is an IncIl plasmid and not an IncF plasmid, as both types of replicons were present in the isolate containing plasmid 15-1.

### WGS of the two ODM reference plasmids and comparison with plasmid databases

To further confirm and extend our findings, the 15-1 plasmid (representing ODM-group A) and the 27-1 plasmid (representing ODM-group D) were subjected to long-read WGS analysis and conjugation experiments. Both plasmids were found to be transferable by conjugation.

WGS data revealed that the *bla*_CTX-M-27_-encoding plasmid 27-1, carried the IncFII/IncFIA/IncFIB replicons with the FAB formula F1:A2:B20. The theoretical barcode from the long-read sequence data was highly similar to the experimental barcode of 27-1 (Fig. 5a). Using the Plasmid Database (PLSDB) online tool^39^, several highly similar plasmids, based on shared hashes (800–910/1000 hashes), were detected from more than one continent (Fig. 5b, Supplementary Table 1) confirming the results obtained by constructing theoretical ODM barcodes from available WGS data. BLAST analyses yielded similar results.

**Figure 6:**
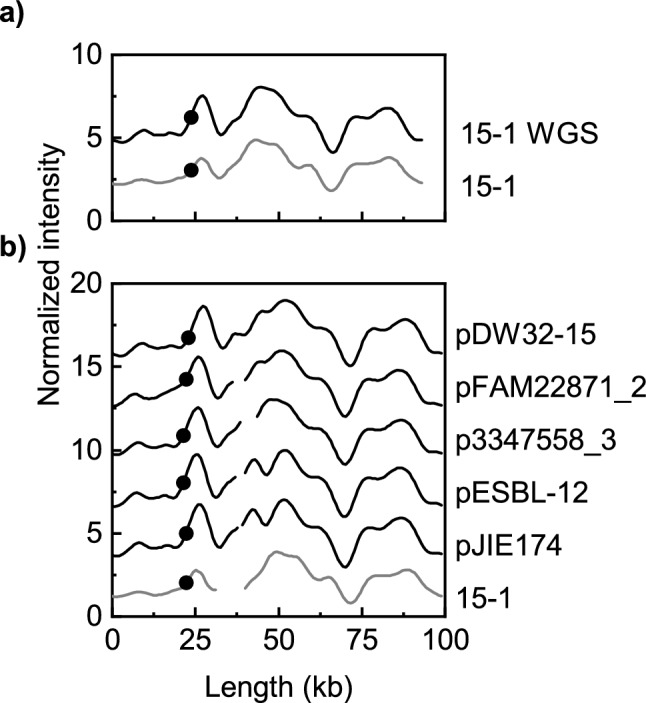
Comparison of ODM-barcodes for the 15-1 plasmid and theoretical barcodes constructed from WGS data. (**a**) Comparison of the experimental ODM barcode from 15-1 with the theoretical barcode constructed from WGS of the 15-1 plasmid. (**b**) Comparison with five highly similar plasmids as detailed in Supplementary Table 2.

WGS analysis of plasmid 15-1 revealed that this was not an IncF plasmid but rather an Incl1 plasmid, more precisely a pMLST37 plasmid. This was also confirmed by COPLA analysis^40^. The plasmid harbored *bla*_CTX-M-15_, as well as AAC(3)-lld (aminoglycoside resistance) and *bla*_TEM-1_ within a 10 kb region, in which *bla*_CTX-M-15_ was flanked by an IS6-like element IS26 family transposase, an IS1380 family transposase upstream, an incomplete cupin fold metalloprotein (WbuC family) and an IS6-like element IS26 family transposase downstream (Supplementary Fig. S3). Four highly similar plasmids (950 out of 1000 shared hashes) were identified by PLSDB. These plasmids were also typed as pMLST37 and harbored the same antibiotic resistance genes (Fig. 6b, Supplementary Table 2). The isolates carrying these plasmids were detected during the same time period as the 15-1 plasmid, in Switzerland^41^, Australia^42^ and Netherlands^43^. The theoretical barcode for plasmid 15-1 from the WGS data was highly similar to the experimental barcode for plasmid 15-1 (Fig. 6a) as were the theoretical ODM-barcodes from the four plasmids identified by WGS analysis, further confirming our findings (Fig. 6b). BLAST analysis identified one more similar plasmid (pDW32-15) in addition to the previous four plasmids.

## Discussion

In this study, we investigated plasmids harboring *bla*_CTX-M_ genes from epidemiologically unrelated and diverse ESBL-*E. coli* strains. The isolates were derived from patients in the hospital as well as from the community setting during a six-year period when these bacteria were gradually established in western Sweden. We have previously successfully used the ODM method for the comparison of epidemiologically related plasmids, including in outbreak investigations^21–24,44^. In this study, we found that the method can also be used for comparing plasmids with the most common *bla*_CTX-M_ genes carried by epidemiologically unrelated isolates. We identified plasmids with both similar and unique ODM-barcodes, for all the *bla*_CTX-M_ genes studied, apart from *bla*_CTX-M-3_ where only plasmids with similar ODM-barcodes were found.

Altogether five groups of plasmids with similar ODM-barcodes were identified, encompassing 57% of the analyzed plasmids. The most prevalent type (ODM-plasmid group A) was found in 28% and the second most prevalent type (ODM-plasmid group D) was found in 11% of the 47 plasmids. Considering that only a fraction of the local ESBL-*E. coli* pool was studied and we aimed for high diversity in the studied isolates rather than focusing on the most prevalent isolate types, this high similarity was a surprising finding. This could imply that a limited number of plasmid types harboring *bla*_CTX-M_ genes were in circulation in western Sweden at the time. Plasmids with similar barcodes generally contained *bla*_CTX-M_ genes from the same CTX-M group, but there was no correlation with a particular healthcare setting, sample source, ST or phylogroup. This further strengthens the hypothesis that HGT was already ongoing in the beginning of the ESBL pandemic in the region, despite being a low-level resistance setting^29^.

Not surprisingly, similarity was observed between the IncF-plasmids (F1:A2:B20) encoding *bla*_CTX-M-27_ detected in the ST131-C1-M27 isolates. This finding is consistent with the described global emergence of the ST131-C1-M27 subclade in association with this plasmid type^11,13,17,32,33^. The varying lengths of the plasmids in this study and published plasmids suggest that insertion/deletions have taken place over time. Considering the global prevalence of ST131-C1-M27 isolates, its more recent global spread and its strong association with F1:A2:B20 plasmids in general, it is not unlikely that the presence of these plasmids (ODM-plasmid group D) is a matter of repeated isolate importation with immigrants or travelers at the time, an often-highlighted transmission route for ESBL-*E. coli* in the early ESBL pandemic in Sweden^18,45^.

Interestingly, a substantial number of isolates carried CTX-M group 1 genes, mostly *bla*_CTX-M-15_, on an IncIl plasmid (ODM-plasmid group A) and not, as mostly described, on IncF plasmids^15^. The barcode and length of these plasmids appear stable over time, with only minor insertions/deletions occurring around the location of the *bla*_CTX-M_ gene, which also varies slightly between plasmids. These plasmids were detected during the entire study period, in isolates of diverse STs and phylotypes, in accordance with the broader host range often described for IncIl plasmids^16^. The heterogeneity of the isolates harboring these plasmids and the rarity of reported IncIl plasmids similar to those in ODM-plasmid group A may further strengthen the likelihood of local HGT for these plasmids. A study investigating *bla*_CTX-M_ encoding plasmids from *E. coli* in a U.S. tertiary care hospital, identified an extensive network of plasmid sharing between isolates of different STs, except for ST131, and especially for a set of IncIl plasmids encoding *bla*_CTX-M-15_^46^. In that study, *bla*_CTX-M-15_ on IncIl plasmids was, however, mostly encoded downstream of ISEcp1 and not IS26 as was the case for the IncIl plasmids we described here.

Plasmids from ODM-plasmid group A were found in isolates belonging to the two high-risk clones ST1193 and ST131, nowadays generally associated with IncF plasmids that appear fixed within the clone or a subclade^8,13,14,47^. However, the global emergence of ST1193 isolates is more recent, and the ST131 isolates containing the ODM-plasmid group A plasmids in this study were part of the early ST131 clades which could explain these findings. The database search revealed isolates with highly similar plasmids already in 2006, which could point to an evolutionarily early plasmid that has not achieved the same success as the IncF plasmids encoding *bla*_CTX-M-15_, unless the apparent rarity of this IncIl plasmid is a result of a lack of reporting. In this respect, it should be noted that all but one isolate carrying a plasmid similar to the IncIl-plasmid also contained IncF replicons, which are considered to be the replicon type mostly associated with *bla*_CTX-M_ genes in human ESBL-*E. coli*^13,15^. We were initially tricked by these replicon-typing results and others could have been misled in the same way, especially in early studies that primarily used replicon typing to characterize plasmids. The literature and relevant databases revealed little information aiding in explaining the presence and possible route of transmission of this IncIl plasmid in western Sweden. The plasmid was found to be transferrable by conjugation and local spread of isolates and plasmids that go undetected is inevitable, especially in the community setting.

We do not believe that a selection bias explains the findings of similar plasmids, considering how dispersed the studied isolates were. On the other hand, due to the limited number of isolates studied, we cannot say that we have described all the plasmids in circulation locally at the time, nor can we estimate their actual prevalence or association with a particular sequence type. Plasmids not sharing similarities with other plasmids may not be unique in a larger context. In addition, extensive rearrangement, that are difficult to identify with ODM, may have taken place as IncF plasmids are reported to be highly plastic^8^.

We did not find it meaningful to include a set of ST131-C2 isolates despite its prevalence in western Sweden already in 2009^30^. The plasmid content in isolates of this clade has been extensively studied, describing a comparatively fixed association and evolution of this clade and its IncF plasmids, also in a study from Sweden^18^. It is thus very likely that these plasmids were present at the time. For the same reason, blood isolates were not included, as ST131 and ST131-C2 in particular, are generally overrepresented among ESBL-*E. coli* bacteremia cases^48^, which could have resulted in a selection bias.

In addition to the epidemiological findings, this study showed that ODM offers a rapid and simple way to analyze and compare plasmids. The study also demonstrates the need of more detailed methods, such as ODM or WGS when comparing plasmid identity, as we have concluded previously^22,23^. WGS requires the more advanced long-read technology to accurately assemble and close plasmid sequences. This requires considerable computational resources and bioinformatics skills, especially if large numbers of plasmids are to be screened. Therefore, as we experienced, reported WGS data on plasmids are often fractioned and not completely closed, making comparisons between studies difficult. However, when data on closed plasmids is available, ODM results can be compared with long-read WGS analyses through theoretical barcodes predicted from the WGS data^28,31^. Another advantage of ODM is that plasmids with similar sequences can be identified despite the presence of relatively large insertions/deletions, as seen, for example, for ODM-plasmid groups B and D.

In conclusion, we found a considerable number of plasmids with similar ODM-barcodes within a set of plasmids from 45 unrelated and disperse isolates collected during 2009–2014. This could indicate that a limited number of plasmids with *bla*_CTX-M_ genes were in circulation and that HGT of these plasmids had taken place already in the early phase of the ESBL pandemic in western Sweden, despite the low levels of antibiotic resistance. This study highlights the importance of local epidemiological surveillance that target both bacterial strains and mobile genetic elements that includes not only well-established clones and plasmids, but also rare strain types. It also highlights the need for temporospatial epidemiological links, for instance, in polyclonal outbreak investigations, so that the detection of a prevalent plasmid could be properly estimated and not overestimated as a potential transmission event. The ODM technique is an easy and valuable method for screening plasmids for similarity. The ability to compare the results with published long-read WGS data is beneficial from an epidemiological perspective.

## Methods

### Patients and isolates

The isolates are part of a large collection of ESBL-*E. coli* strains isolated at the Clinical Microbiology Laboratory, Sahlgrenska University Hospital, Gothenburg, Sweden. The laboratory covered all healthcare units, both in the outpatient and hospital settings in the greater Gothenburg region. No epidemiological relationships were present between the patients contributing isolates in this study.

The *E. coli* isolates were identified according to routine clinical microbiology practices. The disk diffusion method was used for antimicrobial susceptibility testing according to the recommendation of the European Committee on Antimicrobial Susceptibility Testing (EUCAST) (https://www.eucast.org). Cephalosporin-resistant isolates were screened for ESBL-phenotype by the double-disk diffusion assay and ESBL-positive isolates were stored at − 70°C^22^. A large collection of isolates had previously been phylotyped, the CTX-M groups 1, 2 and 9 determined and ST131 isolates including subclade C2 (also named H30Rx) identified as previously described^22,23,49^. From this set of isolates, we arbitrarily selected a subset of isolates aiming for diversity in strain types, covering both urine isolates for diagnostic purposes and fecal screen isolates detected over the entire study period (Tables 1 and 2). We actively excluded isolates part of the ST131 subclade C2. In addition, isolates from this time-period previously subjected to plasmid typing via the ODM technique in two previous studies were included^22,23^. The included isolates were further typed as outlined below.

### Determination of strain types and bla_CTX-M_ genes and replicon types

The frozen isolates were retrieved, plated on blood agar media, and incubated overnight at 37°C. From the bacterial culture, genomic DNA was extracted by boiling method and subsequently subjected to DNA analyses of bacterial strain properties as previously described^49^. The ST131 clades; A, B, C0, C1, C1-M27 and C2 were established by using the PCR assay developed by Matsumura et al.^50^. Multi-locus sequence typing (MLST) was performed to obtain the STs, according to the methods of the MLST database website^51^. Sequencing was performed to determine the specific *bla*_CTX-M_ gene carried by each isolate as described previously^23^. Incompatibility typing of plasmids to determine replicon types was performed by PCR^52^.

### Optical DNA mapping of plasmids

The optical DNA mapping method has been described in detail in previous studies^21,24,26^. Plasmid DNA was prepared from overnight cultures with the NucleoBond Xtra Midi Kit (Macherey–Nagel, Duren, Germany) according to the manufacturer’s instructions for low-copy-number plasmids as previously describe^22^. Subsequently, plasmid samples were treated with Cas9, which locates the resistance gene of interest and linearize it with a double strand cut. The plasmids were then stained by competitive binding between YOYO-1 and netropsin to create a sequence dependent intensity variation along the plasmids and imaged in nanochannels using an inverted epifluorescence microscope. Intensity traces (barcodes) of the plasmids were extracted from the images and used to compare plasmids and identify isolates with similar plasmids. For experimental details see the supplementary information.

To determine if two barcodes from different isolates were similar, a cutoff at a p-value of 0.01 was used in the statistical analysis^24^. In the analysis, we allowed the barcodes to stretch up to 10% to account for minor differences in length and/or stretching in the nanochannels. When preparing the figures, barcodes with similar features were circularly shifted (along the x-axis) to align the features of their barcodes with each other. Gaps were introduced when plasmids of different lengths were compared and illustrate where an insertion/deletion may have occurred in the sequence. The location of the resistance gene is indicated by a black dot, in the cases where the gene location coincided with a gap in the barcode, the dot was placed on the left side of the gap. In the figures the barcodes are shifted along the y-axis (intensity) to avoid overlap and increase readability of the figures.

### WGS of selected plasmids and strains and comparison by theoretical barcode analyses

Genomic DNA was extracted as previously described according to Marmur et al^53^. Long and short read sequencing was performed using nanopore and Illumina generated WGS data as detailed in the supplementary information.

Replicon typing and plasmid MLST were performed using PlasmidFinder 2.1 and pMLST 2.0^19^ from the Center for Genomic Epidemiology (http://www.genomicepidemiology.org), respectively. Antibiotic resistance gene identification analysis was performed using ABRicate v1.0.1^54^, selecting the Comprehensive Antibiotic Resistance Database (CARD)^55^. Previous typing results were confirmed and plasmid MLST data and replicon types were retrieved for the plasmids studied.The two plasmids, 15-1 (representing ODM-plasmid group A) and 27-1 (representing ODM-plasmid group D) were compared to previously published plasmids in WGS databases. Using the respective plasmid sequences as reference, a search was performed using the Plasmid Database (PLSDB)^39^, choosing the search strategy “mash dist”, which is intended to search for plasmids similar to the input sample. Default parameters were used. BLAST analyses were performed using the 15-1 and 27-1 plasmid sequences and the nonredundant nucleotide collection database in GenBank. The number of maximum BLAST hits was set to 5000. COPLA analyses to confirm the Il replicon type was also performed for the 15-1 plasmid (https://castillo.dicom.unican.es/copla). In addition, sequence data for selected plasmids were retrieved from GenBank and theoretical barcodes were constructed for comparison with ODM-barcodes for isolates in the study as previously described^31^.

### Conjugation of plasmids

The strains carrying the *bla*_CTX-M-15_ and *bla*_CTX-M-27_ plasmids 15-1 and 27-1 were subjected to conjugation experiments as outlined in the supplementary information.

### Ethical approval

This study was conducted in accordance with the Declaration of Helsinki and national and institutional standards and was approved by the research ethics committee “Regional Etikprövningsnämnden, EPN” in Gothenburg, Sweden (permit no.170-17).

## Supplementary Information

Supplementary Information.

## Data Availability

The ODM data can be obtained from the corresponding author upon reasonable request. The accession numbers for plasmids 15-1 and 27-1 are CP107236 and CP107231 respectively. The accession numbers for the plasmids included in the theoretical barcode analysis are given in the supplementary tables.
