# Author Correction: Comparison of CTX-M encoding plasmids present during the early phase of the ESBL pandemic in western Sweden

**DOI:** 10.1038/s41598-024-67491-y

**Published:** 2024-07-19

**Authors:** Moa S. Wranne, Nahid Karami, Sriram KK, Daniel Jaén-Luchoro, Shora Yazdanshenas, Yii-Lih Lin, Arpitha Kabbinale, Carl-Fredrik Flach, Fredrik Westerlund, Christina Åhrén

**Affiliations:** 1 Department of Life Sciences, Chalmers University of Technology, Gothenburg, Sweden; 2 Department of Infectious Diseases, Institute of Biomedicine, University of Gothenburg, Guldhedsgatan 10A, 413 46 Gothenburg, Sweden; 3 Department of Clinical Microbiology, Sahlgrenska University Hospital, Region Västra Götaland, Gothenburg, Sweden; 4 Centre for Antibiotic Resistance Research in Gothenburg (CARe), University of Gothenburg, Gothenburg, Sweden; 5 Swedish Strategic Program Against Antimicrobial Resistance (Strama), Region Västra Götaland, Gothenburg, Sweden

Correction to: *Scientific Reports* 10.1038/s41598-024-62663-2, published online 24 May 2024

The original version of this Article contained an error in the name of author Sriram KK, which was incorrectly given as K.K. Sriram.

The original Article has been corrected.

