# Supplementary material for: Comparison of CTX-M encoding plasmids present during the early phase of the ESBL pandemic in western Sweden
Source: Sci Rep. 2024 May 24;14:11880. doi: 10.1038/s41598-024-62663-2 (PMC11126669; doi:10.1038/s41598-024-62663-2)
Supplement: Supplementary file 1 — Supplementary Information. [file 41598_2024_62663_MOESM1_ESM.pdf]

Title: Comparison of CTX-M encoding plasmids present during the early phase of the ESBL pandemic in western Sweden

Authors: Moa S. Wranne, Nahid Karami*, Sriram KK, Daniel Jaén-Luchoro, Shora Yazdanshenas, Yii-Lih Lin, Arpitha Kabbinale, Carl-Fredrik Flach, Fredrik Westerlund, Christina Åhrén[2]

Supplementary information

ODM-method

Plasmids were linearized using CRISPR/Cas9. For targeting *bla*$_{CTX-M}$ group 1 genes crRNA with sequence 5′-CCGTCGCGATGTATTAGCGT-3′ was used and for targeting *bla*$_{CTX-M}$ group 9 genes gRNA 5′-AGAGAGCCGCCGCGATGTGC-3′) was used[1]. gRNA was obtained by mixing equimolar amounts of crRNA and tracrRNA (0.5 nmol each, Dharmacon Inc., Lafayette, CO, USA) in the presence of 1X NEB-3 buffer (New England Biolabs, Ipswich, MA, USA) and 1× bovine serum albumin (BSA, 0.1 µg/mL), and incubating at 4°C for 30 min. To this mixture, Cas9 protein (600 ng, Sigma Aldrich, St. Louis, MI, USA) was added and the sample was incubated at 37°C for 15 min to form Cas9-gRNA complexes. Further, plasmid DNA (60 ng) together with NEB-3 buffer and BSA was added to the tube containing the Cas9–gRNA mixture to a final volume of 30 µl. The mixture was incubated at 37°C for 1 h to let Cas9 linearize the plasmids containing the gene of interest.

In the next step the barcodes were formed by letting netropsin (Sigma-Aldrich) and YOYO-1 (Invitrogen) bind to the plasmids. Plasmid DNA was mixed with λ-DNA (48502 bp, New England Biolabs), used as size reference, YOYO-1 and netropsin in a 1.8:1:70 ratio (DNA:YOYO:netropsin) in 0.5X Tris-Borate-EDTA buffer (TBE, Sigma-Aldrich). The mixture was incubated at 50°C for 30 min. Next, sample together with Milli-Q water (total volume 500 µl) was filtered using a 3 kDa Amicon Ultra-0.5 Centrifugal Filter Unit (Millipore) in a tabletop centrifuge (Eppendorf MiniSpin®) set to 13400 rpm for 17 minutes. The filtrate was removed, and the procedure repeated two more times, the last time with 0.05X TBE with an addition of 2 % (v/v) of β-mercaptoethanol (BME, Sigma-Aldrich) to protect the DNA from photodamage during imaging.

The nanofluidic devices and the fabrication procedure are discussed in detail elsewhere[2,3]. Briefly, the devices consist of four loading reservoirs, with microchannels connecting the reservoirs two and two. The microchannels, in turn, are connected by 200 parallel nanochannels that are 150 nm wide, 100 nm deep, and 500 µm long. The nanochannels enable stretching of the DNA molecules due to the nanoconfinement. The chip was mounted to a chuck, custom-made to fit on top of an epi-fluorescence microscope (Zeiss AxioObserver.Z1). Images were collected using either a 100× oil immersion objective (Zeiss, NA = 1.46), a FITC filter (488 nm excitation/530 nm emission), and an sCMOS camera (Photometrix Prime 95B) or a 63x oil immersion objective (Zeiss, NA=1.46) with an additional 1.6x magnifier, a FITC filter (488 nm excitation/530 nm emission), and an EMCCD camera (Photometrix evolve 512x512 pixels). The chuck was designed with one pressure inlet to each of the four reservoirs, enabling pressure-driven flow of the DNA molecules.

During an experiment, the sample is loaded in one reservoir while the other reservoirs are loaded with buffer. Using pressure, DNA is moved through one of the microchannels and concentrated at the entrance of the nanochannels. With a short pulse of higher pressure, DNA is pushed into the nanochannels where it stretches due to the confinement. An image containing 20 frames (100 ms each) is collected before the DNA is flushed out and a new set of DNA molecules are pushed into the nanochannels.

The images are analyzed using custom-made MATLAB scripts[1]. Each DNA molecule is detected, its length (in µm) is determined and the intensity variation along the molecule obtained. Images for DNA molecules of similar length are grouped together, and the intensity patterns are compared. Identical cut-site among several molecules confirm Cas9 restriction and hence the presence of the resistance gene on the plasmid. If several molecules have the same pattern and Cas9 cut-site, the average pattern is calculated and hereafter called the plasmid's

barcode. λ-DNA (48 502 bp) is used as an internal size reference to retrieve the length of the plasmids in kilo base pairs (kb).

The barcodes from the different plasmids are then pairwise compared to investigate if some are similar. In this comparison we allow up to 10 % stretch of the plasmid length. If the p-value is 0.01 or lower the two barcodes are considered similar[4]. When a group of barcodes ($n \geq 3$) all have p-values that are 0.01 or lower, the barcodes/plasmids are grouped together in an ODM-plasmid group.

When comparing an experimental barcode to WGS data we first create a theoretical barcode from the WGS data, with the help of a custom-made MATLAB script[5]. Then the theoretical barcode and experimental barcode are compared in the same way as described above for two experimental barcodes[6].

Whole-genome sequencing methods

Genomic DNA was extracted as previously described according to Marmur et al[7] DNA samples were quantified with the Qubit® 2.0 fluorimeter and the QubitTM dsDNA BR kit (Thermofisher Scientific, Waltham, MA, USA). Quality was determined by analysis of ratios 260/230 and 260/280 on a NanoDrop ND-1000 spectrophotometer (Thermofisher Scientific, Waltham, MA, USA). Estimation of the distribution of DNA fragment sizes was performed, using a TapeStation 2200 (Agilent Technologies, Santa Clara, CA, USA). DNA samples were sequenced as an external service, using an Illumina NovaSeq 6000 S4 (read mode 2x150 bp) (Eurofins Genomics, Germany). In-house MinION Mk101B long read sequencing (Oxford Nanopore Technologies, United Kingdom) was performed, using a rapid barcoding sequencing kit (SQK-RBK004) and a FLOW-MIN106 vR9.4, during 72 hours on MinKNOWN software v4.3.12 (Oxford Nanopore Technologies, United Kingdom). The reads were thereafter base-

called and demultiplexed using Guppy v6.0.1 (Oxford Nanopore Technologies, United Kingdom) and the quality was determined using NanoPlot v1.32.1[8]. Hybrid assembly of Illumina and nanopore data was performed using Unicycler v0.4.8[9]. Basic quality parameters were determined with the Quality Assessment Tool (QUAST)[10]. The genomes were annotated with the Prokaryotic Genome Annotation Pipeline (PGAP) for submission to GenBank[11].

Conjugation of plasmids

The strains carrying the $bla_{\text{CTX-M-15}}$ and $bla_{\text{CTX-M-27}}$ plasmids 15-1 and 27-1 served as donors and the tetracycline resistant *E. coli* strain CAG18439 served as recipient in filter mating assays. Equal biomasses of the donor and recipient were mixed on 0.22 µm filters placed on Mueller-Hinton agar (MHA). Filters with the respective donor and the recipient alone were prepared as controls. After incubating at 37°C overnight, MHA supplemented with either tetracycline plus cefotaxime or with tetracycline alone was used to select for transconjugants and recipients, respectively. Retrieved transconjugants were checked for carriage of the expected CTX-M gene by PCR.

Accession number	Plasmid name	shared_hashes	BLAST identity	Replicon type	*FAB-formula*	$bla_{CTX-M-27}$	Length (kb)	Species	Reference
NZ_MK295828.1	pU23	910	-	IncFIA, IncFIB, IncFII	F1:A6:B20	YES	122	*E. coli*	[12]
NZ_LC520271.1	pA0140	878	-	IncFIA, IncFIB	F1:A2:B20	YES	120	*E. coli*	[13]
NZ_LC520272.1	pA0145	854	-	IncFIA, IncFIB, IncFII	F1:A2:B20	YES	126	*E. coli*	[13]
CP021871.1	pH105	817	-	IncFIA, IncFIB, IncFII	F1:A2:B20	YES	134	*E. coli*	[14]
NZ_CM017076.1	p-115686	Non available	99,94%	IncFIA,IncFIB	F1:A2:B20	YES	115	*E. coli*	[15]
NZ_CM017099.1	p-116130	Non available	99,95%	IncFIA,IncFIB	F1:A2:B20	YES	116	*E. coli*	[15]

Supplementary Table 1. Reported plasmids highly similar to plasmid 27-1 as identified by the Plasmid Database (PLSDB; 800 /1000 hashes shared,[16] or BLAST analyses.

Accession number	Plasmid name	Shared hashes	BLAST identity	Replicon type	Plasmid pMLST	*bla*$_{CTX-M-15}$	length (kb)	Species	Reference
NZ_EU418931.1	pJIE174	968	-	Incl1	pST37, CC-3	YES	96	*E. coli*	[17]
NZ_CP071076.1	p3347558-3	998	-	Incl1	pST37, CC-3	YES	93	*E. coli*	-
NC_024977.1	pESBL-12	968	-	Incl1	pST37, CC-3	YES	96	*E. coli*	[18]
NZ_KU355874.1	pFAM22871-2	957	-	Incl1	pST37, CC-3	YES	97	*E. coli*	[19]
MK436210.1	pDW32-15	Non available	99,99%	IncI1	pST37, CC-3	YES	98	*E. coli*	-

Supplementary Table 2. Reported plasmids highly similar to plasmid 15-1 as identified by the Plasmid Database (PLSDB; 950 /1000 hashes shared,[16] or BLAST analyses.

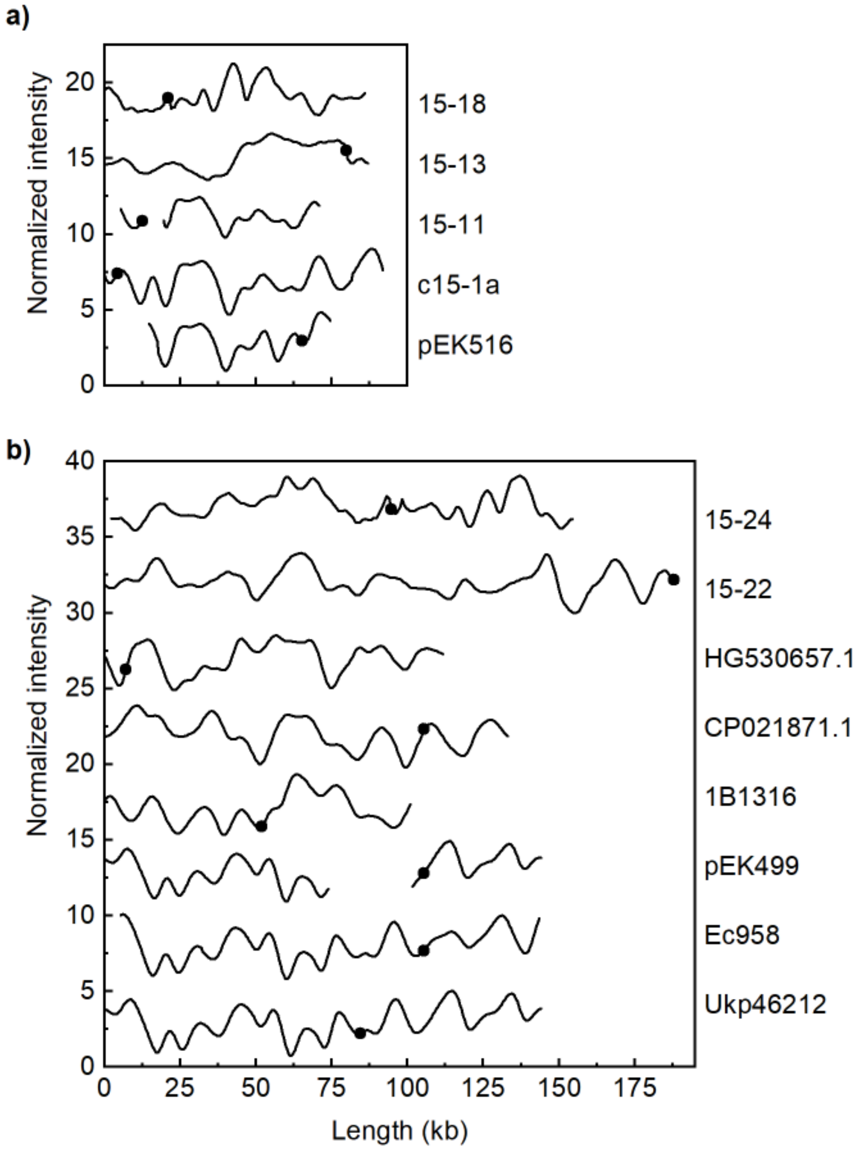

Figure S1. Comparison of ODM-barcodes of *bla*$_{CTX-M-15}$ plasmids (not part of ODM-plasmid group A) from ST131 isolates in this study (15-11, 15-13, 15-18, 15-22 and 15-24) with the theoretical barcode of commonly used IncF references plasmids (c15-1a[20], pEK516[21], Ukp46212[22], Ec958[23] and pEK499[21], and plasmids from another Swedish study from this time period (1B1313, 1CP021871.1 and 1HG530657.1)[24]. **a)** plasmids shorter than 100 kb. **b)** plasmids longer than 100 kb.

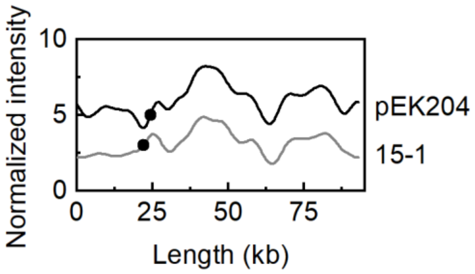

Figure S2. Comparison of the 15-1 plasmid with the theoretical barcode of the pEK204 plasmid described by Woodford et al[21].

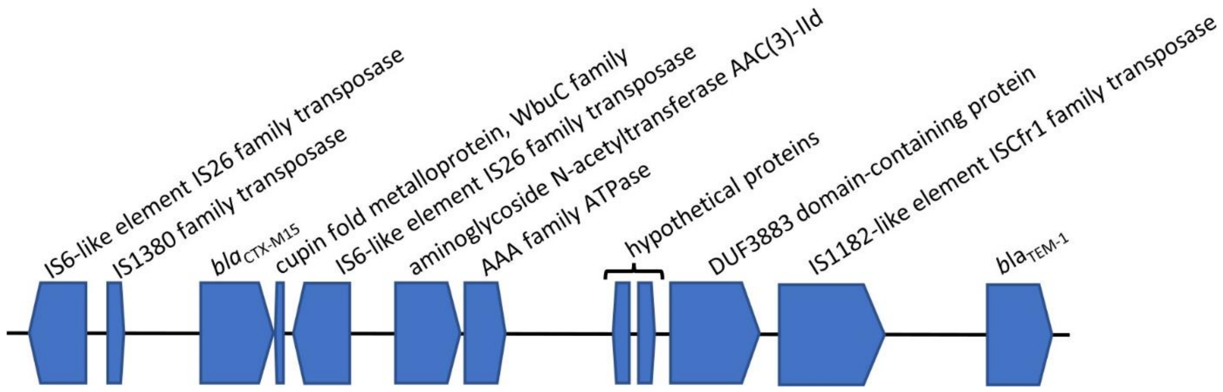

Figure S3. The gene cassette surrounding *bla*$_{\text{CTX-M15}}$ in plasmid 15-1 part of ODM-plasmid group A

References

1. Müller, V. *et al.* Direct identification of antibiotic resistance genes on single plasmid molecules using CRISPR/Cas9 in combination with optical DNA mapping. *Sci Rep* **6**, 37938, doi:10.1038/srep37938 (2016).
2. Frykholm, K., Müller, V., Kk, S., Dorfman, K. D. & Westerlund, F. DNA in nanochannels: theory and applications. *Q Rev Biophys* **55**, e12, doi:10.1017/S0033583522000117 (2022).
3. Kk, S. *et al.* Fluorescence Microscopy of Nanochannel-Confined DNA. *Methods Mol Biol* **2694**, 175-202, doi:10.1007/978-1-0716-3377-9_9 (2024).
4. Müller, V. *et al.* Rapid Tracing of Resistance Plasmids in a Nosocomial Outbreak Using Optical DNA Mapping. *ACS Infect Dis* **2**, 322-328, doi:10.1021/acsinfecdis.6b00017 (2016).
5. Nilsson, A. N. *et al.* Competitive binding-based optical DNA mapping for fast identification of bacteria--multi-ligand transfer matrix theory and experimental applications on *Escherichia coli*. *Nucleic Acids Res* **42**, e118, doi:10.1093/nar/gku556 (2014).
6. Lin, Y. L., Sewunet, T., Kk, S., Giske, C. G. & Westerlund, F. Optical maps of plasmids as a proxy for clonal spread of MDR bacteria: a case study of an outbreak in a rural Ethiopian hospital. *J Antimicrob Chemother* **75**, 2804-2811, doi:10.1093/jac/dkaa258 (2020).
7. Salvà-Serra, F. *et al.* A protocol for extraction and purification of high-quality and quantity bacterial DNA applicable for genome sequencing: a modified version of the Marmur procedure. *Protocolexchange*, doi:https://doi.org/10.1038/protex.2018.08 (2018).
8. De Coster, W., D'Hert, S., Schultz, D. T., Cruts, M. & Van Broeckhoven, C. NanoPack: visualizing and processing long-read sequencing data. *Bioinformatics* **34**, 2666-2669, doi:10.1093/bioinformatics/bty149 (2018).
9. Wick, R. R., Judd, L. M., Gorrie, C. L. & Holt, K. E. Unicycler: Resolving bacterial genome assemblies from short and long sequencing reads. *PLoS Comput Biol* **13**, e1005595, doi:10.1371/journal.pcbi.1005595 (2017).
10. Gurevich, A., Saveliev, V., Vyahhi, N. & Tesler, G. QUAST: quality assessment tool for genome assemblies. *Bioinformatics* **29**, 1072-1075, doi:10.1093/bioinformatics/btt086 (2013).
11. Tatusova, T. *et al.* NCBI prokaryotic genome annotation pipeline. *Nucleic Acids Res* **44**, 6614-6624, doi:10.1093/nar/gkw569 (2016).
12. Kondratyeva, K., Salmon-Divon, M. & Navon-Venezia, S. Meta-analysis of Pandemic *Escherichia coli* ST131 Plasmidome Proves Restricted Plasmid-clade Associations. *Sci Rep* **10**, 36, doi:10.1038/s41598-019-56763-7 (2020).
13. Matsuo, N. *et al.* Characterization of bla(CTX-M-27)/F1:A2:B20 Plasmids Harbored by *Escherichia coli* Sequence Type 131 Sublineage C1/H30R Isolates Spreading among Elderly Japanese in Nonacute-Care Settings. *Antimicrobial agents and chemotherapy* **64**, doi:10.1128/AAC.00202-20 (2020).
14. Ghosh, H. *et al.* Complete Genome Sequence of bla(CTX-M-27)-Encoding *Escherichia coli* Strain H105 of Sequence Type 131 Lineage C1/H30R. *Genome Announc* **5**, doi:10.1128/genomeA.00736-17 (2017).
15. Mostafa, H. H. *et al.* Genomic Surveillance of Ceftriaxone-Resistant *Escherichia coli* in Western New York Suggests the Extended-Spectrum beta-Lactamase bla (CTX-M-27) Is Emerging on Distinct Plasmids in ST38. *Front Microbiol* **11**, 1747, doi:10.3389/fmicb.2020.01747 (2020).
16. Galata, V., Fehlmann, T., Backes, C. & Keller, A. PLSDB: a resource of complete bacterial plasmids. *Nucleic Acids Res* **47**, D195-D202, doi:10.1093/nar/gky1050 (2019).
17. Zong, Z., Ginn, A. N., Dobiasova, H., Iredell, J. R. & Partridge, S. R. Different IncI1 plasmids from *Escherichia coli* carry ISEcp1-blaCTX-M-15 associated with different Tn2-derived elements. *Plasmid* **80**, 118-126, doi:10.1016/j.plasmid.2015.04.007 (2015).
18. Brouwer, M. S. *et al.* Complete Genome Sequences of IncI1 Plasmids Carrying Extended-Spectrum beta-Lactamase Genes. *Genome Announc* **2**, doi:10.1128/genomeA.00859-14 (2014).

19. Marti, R. *et al.* Short communication: Heat-resistant *Escherichia coli* as potential persistent reservoir of extended-spectrum beta-lactamases and Shiga toxin-encoding phages in dairy. *J Dairy Sci* **99**, 8622-8632, doi:10.3168/jds.2016-11076 (2016).
20. Boyd, D. A. *et al.* Complete Nucleotide Sequence of a 92-Kilobase Plasmid Harboring the CTX-M-15 Extended-Spectrum Beta-Lactamase Involved in an Outbreak in Long-Term-Care Facilities in Toronto, Canada. *Antimicrobial agents and chemotherapy* **48**, 3758-3764, doi:10.1128/AAC.48.10.3758-3764.2004 (2004).
21. Woodford, N. *et al.* Complete nucleotide sequences of plasmids pEK204, pEK499, and pEK516, encoding CTX-M enzymes in three major *Escherichia coli* lineages from the United Kingdom, all belonging to the international O25:H4-ST131 clone. *Antimicrobial agents and chemotherapy* **53**, 4472-4482, doi:10.1128/AAC.00688-09 (2009).
22. Stoesser, N. *et al.* Evolutionary History of the Global Emergence of the *Escherichia coli* Epidemic Clone ST131. *mBio* **7**, e02162, doi:10.1128/mBio.02162-15 (2016).
23. Forde, B. M. *et al.* The complete genome sequence of *Escherichia coli* EC958: a high quality reference sequence for the globally disseminated multidrug resistant *E. coli* O25b:H4-ST131 clone. *PloS one* **9**, e104400, doi:10.1371/journal.pone.0104400 (2014).
24. Ny, S., Sandegren, L., Salemi, M. & Giske, C. G. Genome and plasmid diversity of Extended-Spectrum beta-Lactamase-producing *Escherichia coli* ST131 - tracking phylogenetic trajectories with Bayesian inference. *Sci Rep* **9**, 10291, doi:10.1038/s41598-019-46580-3 (2019).
